# Impact of SiO_2_ Particles in Polyethylene Textile Membrane for Indoor Personal Heating

**DOI:** 10.3390/nano10101968

**Published:** 2020-10-04

**Authors:** Mohamed Boutghatin, Salim Assaf, Yan Pennec, Michèle Carette, Vincent Thomy, Abdellatif Akjouj, Bahram Djafari Rouhani

**Affiliations:** Institute of Electronic, Microelectronic and Nanotechnology (IEMN), Université de Lille, 59655 Villeneuve d’Ascq, France; mohamed.boutghatin@univ-lille.fr (M.B.); salim.alhajj-assaf@univ-lille.fr (S.A.); michele.carette@univ-lille.fr (M.C.); vincent.thomy@univ-lille.fr (V.T.); abdellatif.akjouj@univ-lille.fr (A.A.); bahram.djafari-rouhani@univ-lille.fr (B.D.R.)

**Keywords:** textile membrane, polyethylene, SiO_2_ particles, mid-infrared, heating, thermal comfort

## Abstract

Keeping the human body in a thermal comfort state inside a room has become a challenge in recent years. While the most common strategy is to heat buildings, it requires a lot of energy. Reducing this energy consumption will have positive impacts, both economically and environmentally. We propose here to act directly on the personal thermal heating of the human body, by modulating the absorption and transmission properties of a synthetic polymer membrane in the mid-infrared (MIR). We show numerically that 5% SiO_2_ submicron particles inserted in polyethylene (PE) and nanoporous polyethylene (nanoPE) membranes increase the radiative heating of the membrane, reducing the required ambient temperature of a room by more than 1.1 °C. The proposed membrane can be flexible enough to be easily integrated into conventional textiles.

## 1. Introduction

The reduction of energy consumption has become a societal challenge that has stimulated research in many directions in the scientific community. As a matter of fact, for indoor areas, about 50% of a building’s energy consumption is attributed to temperature regulation [1]. To reduce this energy consumption, a new approach, called “personal thermal management”, has been proposed in recent years [2,3,4]. This strategy consists of controlling the temperature of the space between the human skin and the textile, called microclimate (mc), rather than regulating the temperature of the entire residential space. In that context, many efforts have been made to develop radiative cooling and heating textiles.

For personal cooling, a textile fabric, made of structured synthetic polymer fibers, which maximizes mid-infrared (MIR) transmission while maintaining opaqueness in the visible range, has been proposed [2]. Hsu et al. [3] demonstrated experimentally that nanoporous polyethylene (nanoPE) can be a good candidate for the cooling purpose. The embedding of nanopores into PE microfibers not only ensures visible opacity without compromising the mid-infrared transparency, but it also achieves cotton-like softness [4]. Conversely, to warm the human body, we showed for the first time that photonic crystal structuration of a synthetic polymer was able to increase the temperature of the human body [5]. For an appropriate set of geometric parameters, we found that the superior heating effect resulted in a 1 °C drop in the ambient temperature setting point. To extend the temperature adaptability to human skin, Hsu et al. [6] demonstrated the ability of a reversible asymmetrical bilayer membrane to achieve dual thermal functionality (heating and cooling), each layer being characteristic of a specific emissivity in the infrared. Dual thermal functionality has also been demonstrated by taking advantage of the thermal effect of temperature-sensitive shape-memory polymers [7]. This dual control has been also studied in the geometrical adaptation of yarns [8], with a dynamic response depending on the temperature and/or humidity.

Another strategy to control radiative cooling and heating is to insert nanotubes or particles of different materials inside a matrix. To optimize the infrared emissivity of polymer fibers, the use of carbon nanotubes was investigated [9]. Integration of particles in a polymer matrix has been used before to increase emissivity in the MIR for daytime radiative cooling of materials and substrates [10,11,12]. For example, a coating composed of TiO_2_ and SiO_2_ particles on a reflective substrate demonstrated excellent selective emission properties for the purpose of radiative cooling [11]. In the textile context, Cai et al. [13] studied a spectrally selective nanocomposite, using 500 nm diameter zinc oxide (ZnO) particles embedded in PE for outdoor personal cooling. They showed that, because of the ZnO particles, such textile reflects more than 90% of the solar irradiance while selectively transmitting out human body thermal radiation. For heating function, a textile covering made of metallic nanowires has been proposed, where heat is produced by the reflectivity and Joule effects in stimulating the metallic nanowires [14]. Until now, the effect of non-metallic particle insertion into a synthetic polymer membrane for personal radiative heating has not been reported.

In the present paper, we aim to demonstrate the effect of SiO_2_ particles embedded in a PE membrane for heating functionality. This thermoplastic polymer, used in a wide range of applications, is textile compatible and intrinsically transparent in the MIR [15]. Our objective is to take advantage of the absorption of electromagnetic waves in the MIR, due to the presence of the particles, to increase the human body temperature. We show that improvement in radiation heating results from a balance between the absorption and transmission of the membrane as a function of the particles’ volume fraction. After presenting the model and method, we propose to study the effect of inserting SiO_2_ particles in a PE membrane on the optical and thermal responses. We then consider the effect of the thickness of the membrane in the absorption mechanism. Optimization of a nanoporous PE membrane is finally proposed, considering the thickness and the volume fraction of SiO_2_ particles, to provide a heating textile-compatible membrane that is flexible and water wicking.

## 2. Model and Method

**Figure 1:**
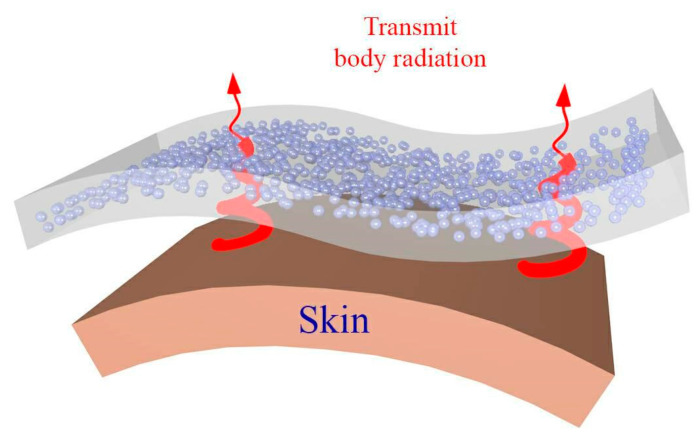
Schematic representation of the ${\mathrm{SiO}}_{2}$ particles embedded in a polyethylene (PE) membrane, designed for radiative indoor heating. The transmitted thermal radiation from the human body is affected by the ${\mathrm{SiO}}_{2}$ particle absorption.

The introduction of particles smaller than the wavelength of the incident electromagnetic wave (in this study, comprised between 5 and 15 µm) in a polymer matrix modifies the complex refractive index n(λ)+jk(λ) of the polymer, where n(λ) and k(λ) are, respectively, the real and imaginary parts of the refractive index, and $j=\sqrt{-1}$. To reach the objective of increasing the electromagnetic absorption of PE in the MIR, we were interested in varying the imaginary part k(λ) of the refractive index. Silicon dioxide (SiO_2_) is characterized by a strong absorption peak around the wavelength of 9 µm, due to an asymmetrical stretching of the molecule, and a smaller peak around 12 µm, resulting from symmetrical stretching [16,17]. With the insertion of SiO_2_ particles, we expected a modulation in the nanocomposite membrane absorption, leading to regulation of thermal properties, as illustrated schematically in Figure 1.

**Figure 2:**
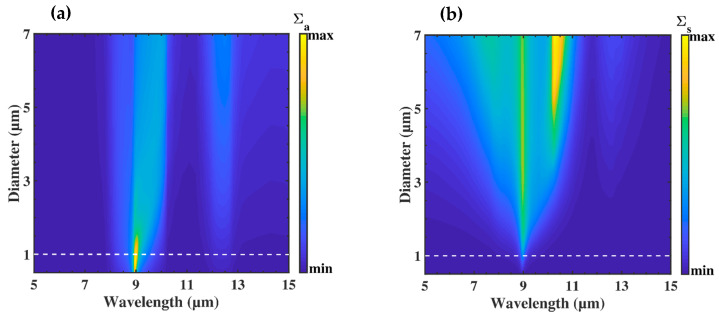
Normalized (**a**) absorption cross-section $\sigma_{a}$ and (**b**) scattering cross-section $\sigma_{s}$ of a single ${\mathrm{SiO}}_{2}$ particle in PE medium over the wavelength range 5–15 μm as a function of the diameter *D* of the particle, varied from 0.5 to 7 μm.

To estimate the appropriate size of a spherical SiO_2_ particle embedded in the PE medium, we first calculated, using Mie theory [18], the scattering limit of a single particle in the wavelength range 5–15 µm. Figure 2 shows the evolution of the normalized absorption and scattering cross-sections $\sum_{a,s}(\frac{\sigma_{a,s}}{\pi (D/2)})$ as a function of the diameter *D* of the particle, where $\sigma_{a,s}$ are, respectively, the absorption and scattering cross-sections. When the diameter was lower than 1 µm (Figure 2a), a strong absorption peak appeared around λ = 9 µm and a weak one around λ = 12 µm. When the diameter was larger than 1 µm (Figure 2b), a scattering effect occurred around λ = 9 µm for diameters above 1 µm and a second one at λ = 11 µm for particles with diameters larger than 5 µm.

This means that for diameters smaller than 1 µm, the scattering effects can be neglected, and that polymer coated with these submicron particles can be considered as a homogeneous medium, defined with effective optical properties. Considering that submicron particles are far from each other and are randomly and uniformly distributed, we used the effective medium theory to calculate the refractive index of the nanocomposite [19,20]. Among the effective theories, Maxwell Garnett (MG) and Bruggeman’s (BG) models gave nearly the same results. We chose the BG model, which offered the possibility to extend the calculation to multiple different media. The optical effective properties $\varepsilon_{\mathrm{BG}}$ of the nanocomposite were then obtained from the resolution of the following equation [21,22]:

(1) $$f_{\mathrm{PE}}\frac{\varepsilon_{\mathrm{PE}}-\varepsilon_{\mathrm{BG}}}{\varepsilon_{\mathrm{PE}}-2\varepsilon_{\mathrm{BG}}}+f_{{\mathrm{SiO}}_{2}}\frac{\varepsilon_{{\mathrm{SiO}}_{2}}-\varepsilon_{\mathrm{BG}}}{\varepsilon_{{\mathrm{SiO}}_{2}}-2\varepsilon_{\mathrm{BG}}}=0$$

where $f_{i}$ denotes the volume fraction of component i, $\sum_{i}\mathrm{fi}=1$, and $\varepsilon_{\mathrm{PE}}$ and $\varepsilon_{{\mathrm{SiO}}_{2}}$ are the dielectric permittivity of PE and SiO_2,_ respectively. From Equation (1), one can see that the effective medium permittivity is dependent on $f_{{\mathrm{SiO}}_{2}}$, which is at the origin of different spectral responses of the membrane to an incident electromagnetic wave. To calculate the reflection (R) and transmission (T) of the effective medium in the MIR, we used the generalized transfer-matrix method [23]. The absorption coefficient was then deduced following the equation A = 1 – R – T. 

## 3. Results and Discussion

**Figure 3:**
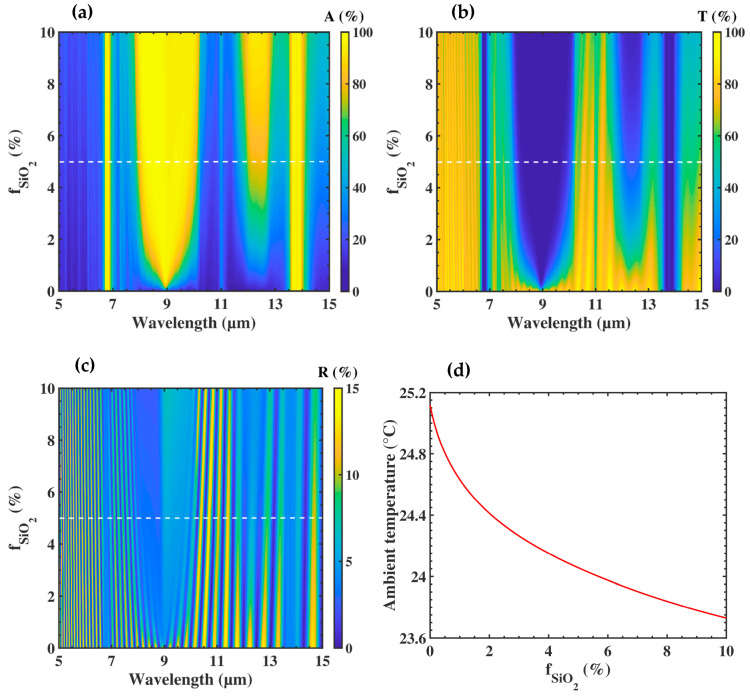
Evolution of (**a**) the absorption (A), (**b**) transmission (T), and (**c**) reflection (R), as a function of the volume fraction of ${\mathrm{SiO}}_{2}$. submicron particles over the wavelength 5–15 µm. (**d**) Evolution of the ambient temperature required by the skin to achieve $T_{s}=34{}^{\circ}C$, as a function of the volume fraction of ${\mathrm{SiO}}_{2}$ submicron particles.

In order to show the effect of submicron particles on the optical response of the membrane, we fixed the thickness of the PE membrane, and we gradually increased the volume fraction of the submicron particles. In the literature, PE films 250 µm thick are used in flexible solar panel applications [24]. For textiles, the thickness of polymer films used for coating surfaces is usually up to $h_{\mathrm{PE}}$ = 100 µm [25]. The three coefficients R, T, and A were calculated as a function of $f_{{\mathrm{SiO}}_{2}}$ for $h_{\mathrm{PE}}$ = 100 µm, under normal incidence (Figure 3). As we can see, the fraction of submicron particles influenced the absorption and transmission spectra, while the average reflection was almost constant and remained lower than 10%. Looking at the absorption spectrum (Figure 3a), four vertical absorption bands appeared with maxima, respectively, close to 7, 9, 12, and 14 µm. We calculated the complex refractive index of the nanocomposite, deduced from $n(\lambda )+\mathrm{jk}(\lambda )=\sqrt{\varepsilon_{\mathrm{BG}}(\lambda )}$, for a filling fraction $f_{{\mathrm{SiO}}_{2}}$ = 5% (see Supplementary Information SI-1). The four absorption bands corresponded to the extinction peaks of the (SiO_2_)-embedded PE membrane in the MIR. Two of them (9 µm and 12 µm) increased with the filling fraction of the submicron particles and were identified as the molecular absorption of the SiO_2_. The two others (7 µm and 14 µm) were independent of the filling fraction of the submicron particles and corresponded to intrinsic vibrations of the PE macromolecules. One can note that, between $f_{{\mathrm{SiO}}_{2}}=0\%$ (PE without submicron particles) and $f_{{\mathrm{SiO}}_{2}}=10\%$, the average amplitudes of A and T, respectively, increased and decreased by more than 40%.

To analyze the thermal properties of the nanocomposite membrane, we used a one-dimensional heat transfer model (see Supplementary Information SI-2). In the frame of this model, the ingoing and outgoing heat flows were studied through two control volumes, the first one around the human body and the second one comprising the membrane and the surrounding air. A third equation was written by considering the thermal conduction inside the membrane. The thermal balance thus gives rise to three equations. To solve this set of equations, the skin temperature was fixed to 34 °C, which corresponds to the upper usual comfort temperature of the human body. We then determined the three unknown temperatures: at the inner (*T_i_*) and outer (*T_o_*) surfaces of the membrane, and in the ambient air (*T_a_*), varying the filling fraction $f_{{\mathrm{SiO}}_{2}}$.

**Figure 4:**
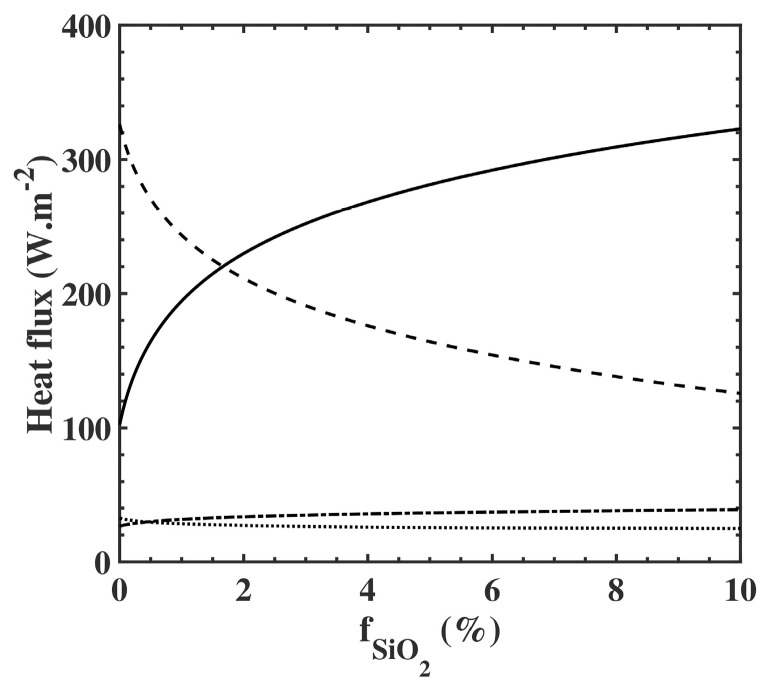
Representation of the radiated flux from the membrane (*m*), (*Q_rad,m_*, black solid line), the transmitted flux from the ambient air (*a*) ($\tau .Q_{\mathrm{rad},a}$, dashed lines), the reflected flux on the membrane toward the skin (*s*) ($\rho .Q_{\mathrm{rad},s}$, dotted lines), and the conducted flux through the microclimate (*mc*) ($Q_{\mathrm{cond},\mathrm{mc}}$, dash-dotted lines), as a function of the volume fraction of ${\mathrm{SiO}}_{2}$ submicron particles. *τ* and *p* are the average transmission and reflection coefficients, defined in Supplementary Information.

Due to the extreme thinness of the membrane, we obtained almost *T_i_* = *T_o_*. Figure 3d shows the evolution of the required ambient temperature (*T_a_*) to achieve the comfort skin temperature of *T_s_* = 34 °C as a function of the filling fraction. When the membrane was free of particles ($f_{{\mathrm{SiO}}_{2}}=0\%$), we found an ambient temperature of *T_a_* = 25.1 °C. When $f_{{\mathrm{SiO}}_{2}}$ increased to 10%, *T_a_* decreased down to 23.7 °C. To understand this behavior, we drew in Figure 4 the evolutions of the flux which contributed to the thermal exchange at the surface of the skin. All these flows were involved in Equation (S1) given in SI-2. When we increased the SiO_2_ volume fraction, the only flows which presented a substantial variation were the transmitted one from the ambient air (*a*) ($\tau .Q_{\mathrm{rad},a}$, dashed lines, where τ is the average transmission, defined in Supplementary Information), and the radiated one from the membrane (*m*), (*Q_rad,m_*, black solid line). As the ambient temperature decreased, the former flux decreased due to a lower membrane transmission, whereas the latter flux increased due to a higher membrane absorption. The heating compensation came from the radiative heat flux, due to the higher emissivity $\varepsilon_{m}$ of the membrane.

We found that increasing the filling fraction of submicron particles in the PE matrix allowed to decrease the room temperature needed for the thermal comfort. Nevertheless, to prevent embrittlement of the composite, and therefore improve the mechanical flexibility of the textile, it was better to choose a low filling factor $f_{{\mathrm{SiO}}_{2}}$. As seen in Figure 3a, a good compromise could be 5% SiO_2,_ which offers efficient absorption of more than 35% of the nanocomposite, as compared to the free membrane.

**Figure 5:**
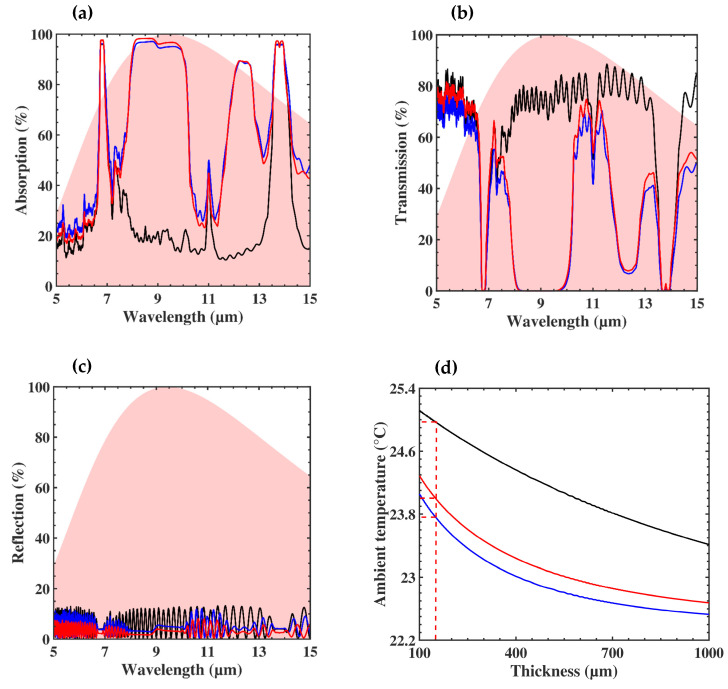
(**a**) Absorption, (**b**) transmission, and (**c**) reflection spectra in the mid-infrared (MIR) for membrane thickness of 150 µm. The black curves correspond to the PE membrane free of submicron particles, the blue and red ones to the PE and nanoPE membranes containing 5% ${\mathrm{SiO}}_{2}$ submicron particles, respectively. The shaded area corresponds to the human body radiation at 34 °C. (**d**) Evolution of the required ambient temperature to achieve thermal comfort $T_{S}=34\;{}^{\circ}C$ as a function of the thickness of the three membranes.

In what follows, we proposed to estimate the effect of the thickness of the membrane on the optical and thermal properties. Figure 5d shows the evolution of the required ambient temperature *T_a_* to keep the thermal comfort (*T_s_* = 34 °C) as a function of the thickness of the free membrane (black solid line) and the 5% composite one (blue solid line). When the membranes became thicker, the required ambient temperature decreased in both cases, with a systematic lower *T_a_* for the 5% SiO_2_-PE membrane. The highest difference in temperature between the two membranes was reached near $h_{\mathrm{PE}}=400\;µm$ (1.3 °C), while for $h_{\mathrm{PE}}=100\;µm$ the difference was 1 °C.

Nevertheless, we needed to reconcile an increase in thickness for higher efficiency and a flexibility of the membrane for textile applications. Previous studies have shown that the introduction of nanoscale pores in PE fibers modifies their mechanical hardness, giving them a cotton-like softness [4]. As reported in the literature [13], a nanoPE membrane with a thickness of $h_{\mathrm{nanoPE}}=150\;µm$, with $f_{\mathrm{air}}=20–30\%$ of nanopores (porosity), is a good compromise in textile applications. We thus proposed a design considering the insertion of 5% SiO_2_ submicron particles in a 25% nanoPE membrane. To calculate the corresponding effective refractive index, we added the term corresponding to nanopores (air) in Equation (1) and solved the following Equation (2):

(2) $$f_{\mathrm{PE}}\frac{\varepsilon_{\mathrm{PE}}-\varepsilon_{\mathrm{BG}}}{\varepsilon_{\mathrm{PE}}-2\varepsilon_{\mathrm{BG}}}+f_{{\mathrm{SiO}}_{2}}\frac{\varepsilon_{{\mathrm{SiO}}_{2}}-\varepsilon_{\mathrm{BG}}}{\varepsilon_{{\mathrm{SiO}}_{2}}-2\varepsilon_{\mathrm{BG}}}+f_{\mathrm{air}}\frac{\varepsilon_{\mathrm{air}}-\varepsilon_{\mathrm{BG}}}{\varepsilon_{\mathrm{air}}-2\varepsilon_{\mathrm{BG}}}=0$$

where $\varepsilon_{\mathrm{air}}$ is the dielectric permittivity of air.

Figure 5a–c shows the evolution of, respectively, the absorption, transmission, and reflection in the MIR for a PE membrane (i) free of submicron particles (black curve), containing 5% SiO_2_ embedded in (ii) PE (blue curve), and (iii) nanoPE (red curve). The thickness of the membranes was $h_{\mathrm{PE}}=150\;µm$. As mentioned previously, one can see two peaks of absorption around 7 µm and 14 µm, attributed to the PE matrix. The insertion of SiO_2_ submicron particles gave rise to broad absorption bands around 9 μm and 12 μm, leading to prohibited bands in the transmission spectrum. The introduction of nanopores inside the PE almost did not affect the optical responses, and the two composite membranes absorbed the majority of human body radiation (shaded area). Regarding the effect of temperature, Figure 5d shows a slight increase of 0.2 °C in the room temperature when the nanopores were introduced into the PE matrix (red curve compared to the blue one). For example, to keep the skin temperature at 34 °C with the thickness of the membrane *h* = 150 µm, we needed *T_a_* = 25 °C for the PE free of submicron particles, while when the membrane contained 5% SiO_2_ submicron particles, the *T_a_* dropped to 23.8 °C and 24 °C, respectively, for the PE and nanoPE. The main advantage of this latter method was to introduce mechanical flexibility that had a low impact on the required room temperature.

In the visible range, ${\mathrm{SiO}}_{2}$ submicron particles did not modify the optical properties of the PE matrix, as both materials had a similar refractive index in the range 0.3–0.7 µm [21,22]. For this reason, the PE transparency in the visible range will not be affected by the insertion of submicron particles. However, nanopores significantly changed the transparency of $5\%\;{\mathrm{SiO}}_{2}$-PE in the visible range [3]. The size of the nanopores and the contrast of the refractive index between PE and air allowed light to diffuse. Therefore, the proposed nanoPE-based membrane is opaque in the visible range. 

**Figure 6:**
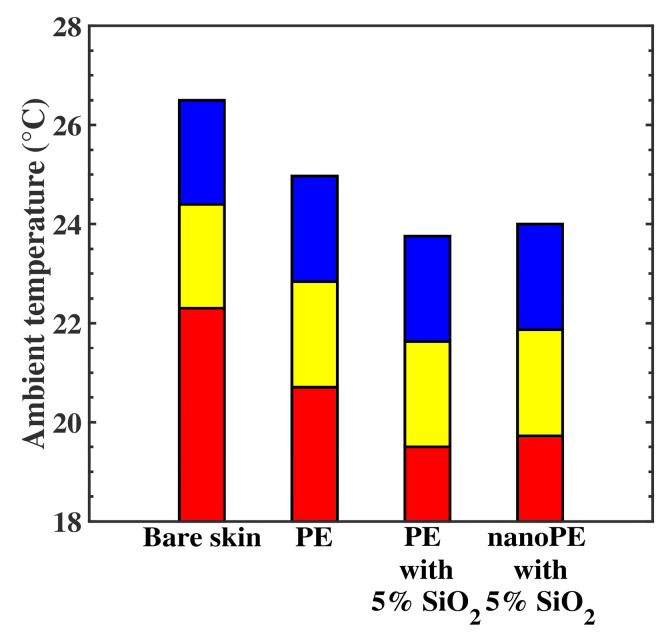
Room temperature for thermal comfort when the human body is covered with PE membrane free of submicron particles, PE membrane containing 5% SiO_2_, and nanoPE membrane containing 5% SiO_2_, compared to bare skin. The thickness of the membranes is 150 µm. The calculation was done for three skin temperatures of thermal comfort, *T_s_* = 30 °C (**red**), *T_s_* = 32 °C (**yellow**), and *T_s_* = 34 °C (**blue**).

Many subjective parameters are attached to thermoregulation of the human body, linked to the apparent temperature and personal resentment [26], leading to skin temperatures ranging from 30 °C to 34 °C. The three membranes, i.e., PE free of submicron particles, PE containing 5% SiO_2_, and nanoPE containing 5% SiO_2_, have been tested (Figure 6) at the three significant temperatures of thermal comfort, i.e., *T_s_* = 30 °C (Figure 6, red), *T_s_* = 32 °C (yellow), and *T_s_* = 34 °C (blue). The thickness of the three membranes was 150 µm.

For each membrane, we calculated the required room temperature to reach *T_s_* and then compared the result with the one obtained for bare skin. Figure 6 shows that, to reach the thermal comfort, the room could be heated from 20 °C to 24 °C when the body was covered with the nanocomposite membranes. In comparison, the required room temperature was systematically 1.1 °C higher for the PE membrane free of submicron particles and 2.5 °C higher for bare skin.

**Figure 7:**
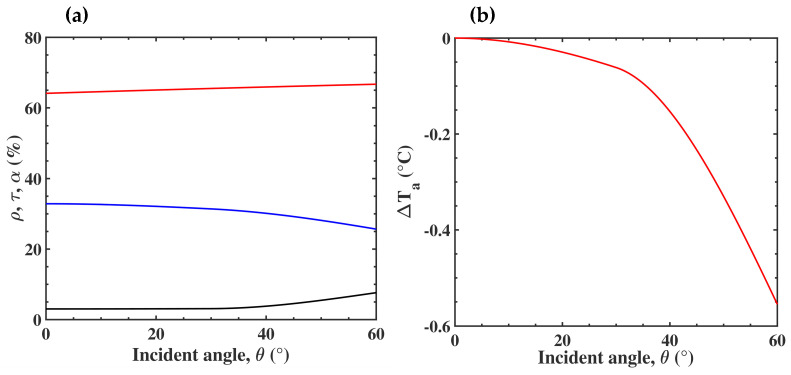
(**a**) Angular dependence of normalized reflectance ρ (black), transmittance τ (blue), and absorbance α (red) in the MIR. (**b**) Evolution of the required ambient temperature to keep *T_s_* = 34 °C, as a function of the incident angle, θ.

All previous calculations were done considering a plane wave under normal incidence. Nevertheless, in reality, partly due to the formation of folds in the garment, the waves can have different angles of incidence. We then calculated the optical response of the 5% SiO_2_ submicron particles embedded in nanoPE under a variation of the incidence angle, *θ*. To simulate the unpolarized wave, the results were presented by taking the average of the two polarizations, i.e., transverse electric (TE) and transverse magnetic (TM). Figure 7a shows the evolution of the efficient optical coefficients *ρ*, *τ*, and *α* as a function of *θ*. The expressions of these coefficients are reported in SI-2. The normalized absorbance *α* increased slightly, but the main effect was an increase of the reflection from 3% to 8% for *θ* varying between 40° and 60°, and a subsequent decrease in the transmission. As before, we calculated the room temperature to get *T_s_* = 34 °C, associated to these coefficients (Figure 7b). We found that, beyond 40° of incidence, the ambient temperature could be decreased by 0.5 °C, compared to the normal incidence, giving an additional benefit to the composite membrane.

## 4. Conclusions

We investigated the effect of SiO_2_ submicron particles in a PE matrix on the optical and thermal properties. We demonstrated that the insertion of 5% SiO_2_ submicron particles produced a significantly higher absorbance in the MIR. In consequence, the higher emissivity of the membrane increased the thermal insulation, allowing a decrease of the room temperature by 1.1 °C. Additionally, the incident angle produced a higher reflectance, which was also in favor of the thermal insulation, up to 0.5 °C. Moreover, embedding the SiO_2_ in a nanoPE matrix offered the necessary breathability and flexibility of a textile without significantly changing the thermal properties. We demonstrated that such membranes, integrated into a textile, could save 1 °C on room heating without compromising personal thermal comfort.

## Supplementary Materials

The following are available online at https://www.mdpi.com/2079-4991/10/10/1968/s1, SI-1: Variation of the ${\mathrm{SiO}}_{2}$-PE refractive index, SI-2: Heat transfer model.
