# Supplementary material for: Impact of SiO2 Particles in Polyethylene Textile Membrane for Indoor Personal Heating
Source: Nanomaterials (Basel). 2020 Oct 4;10(10):1968. doi: 10.3390/nano10101968 (PMC7599470; doi:10.3390/nano10101968)
Supplement: Supplementary file 1 [file nanomaterials-10-01968-s001.pdf]

nanomaterials

Supplementary information

Impact of SiO_2 Particles in Polyethylene Textile Membrane for Indoor Personal Heating

Mohamed Boutghatin, Salim Assaf, Yan Pennec *, Michèle Carette, Vincent Thomy, Abdellatif Akjouj and Bahram Djafari Rouhani

Institute of Electronic, Microelectronic and Nanotechnology (IEMN), Université de Lille, 59655 Villeneuve d'Ascq, France; mohamed.boutghatin@univ-lille.fr (M.B.); salim.alhajj-assaf@univ-lille.fr (S.A.); michele.carette@univ-lille.fr (M.C.); vincent.thomy@univ-lille.fr (V.T.); abdellatif.akjouj@univ-lille.fr (A.A.); bahram.djafari-rouhani@univ-lille.fr (B.D.R.)

* Correspondence: yan.pennec@univ-lille.fr; Tel.: +320436807

SI-1: Variation of the SiO_2-PE refractive index

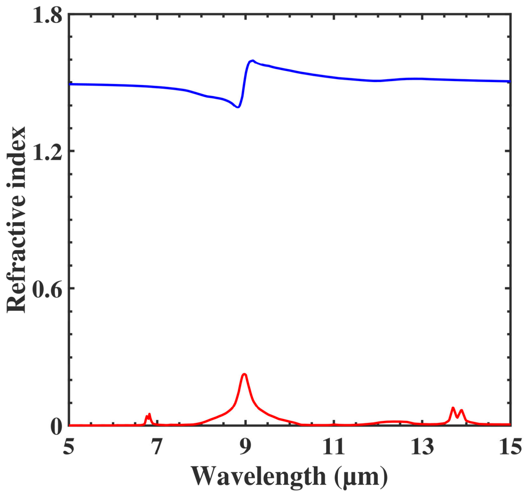

Figure S1. The real (blue) and the imaginary (red) part of the effective refractive index for SiO_2-PE when $f_{SiO_2} = 5\%$. The calculation is done using Bruggeman's (BG) model.

SI-2: Heat transfer model

To quantify the impact of SiO_2-submicron particles on the personal thermoregulation in indoor space, a heat balance has been done between the human body and the surrounding environment. To this aim, we used a one-dimensional steady-state heat transfer model [1]. In our case, we considered a middle-aged man standing up and relaxed with a metabolic heat generation corresponding to $Q_g = 70W\ m^{-2}$ [2]. For a textile-covered skin, heat exchange is described by the following three equations:

Around the human body

$$Q_g + Q_{rad,m} + \tau.Q_{rad,a} - (1-\rho).Q_{rad,s} - Q_{cond,mc} = 0 \quad (1)$$

Around the membrane

$$(1-\rho-\tau).Q_{rad,s} + (1-\rho-\tau).Q_{rad,a} + Q_{cond,mc} - 2.Q_{rad,m} - Q_{conv} = 0 \quad (2)$$

Inside the membrane (at the outer surface)

$$T_o = \frac{h_m}{2k_m}\left(2\varepsilon_m\sigma\left(\frac{T_i + T_o}{2}\right)^4 - \alpha\sigma T_s^4 - \alpha\sigma T_a^4\right) - \frac{k_a h_m}{k_m h_{mc}}(T_s - T_i) + T_i \tag{3}$$

For bare skin, the heat balance is expressed as:

$$Q_g + Q_{rad,a} - Q_{rad,s} - Q_{conv} = 0 \tag{4}$$

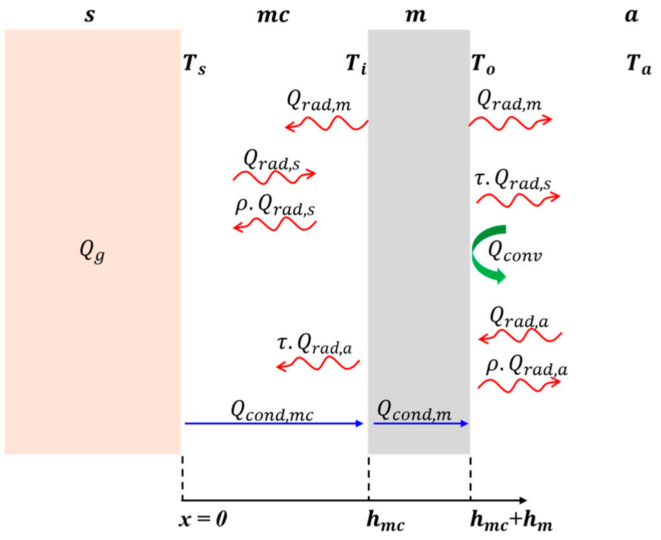

Figure S2. Schematic representation of the heat transfer model where *s* is the skin, *mc* the microclimate, *m* the membrane, and *a* the air.

Where $Q_{rad,m}$ is the radiative heat flux from the membrane, $Q_{rad,a}$is the radiative heat flux from the environment, $Q_{rad,s}$ is the radiative heat flux from the skin, $Q_{cond,mc}$ is the conductive heat flux inside the microclimate between the skin and the membrane inner surface and Q_{conv} is the convective heat flux from the membrane to the ambient air. The heat flux can be expressed as:

$$Q_{rad,m} = \varepsilon_m\sigma\left(\frac{T_i + T_o}{2}\right)^4 \tag{5}$$

$$Q_{rad,a} = \varepsilon_a\sigma T_a^4 \tag{6}$$

$$Q_{rad,s} = \varepsilon_s\sigma T_s^4 \tag{7}$$

$$Q_{cond,mc} = k_a\frac{T_s - T_i}{h_{mc}} \tag{8}$$

$$Q_{conv} = h_c(T_o - T_a) \tag{9}$$

Where T_s is the temperature of the skin, T_a is the temperature of the environment, T_i and T_o are respectively the inner and outer surfaces membrane temperatures, k_a is the thermal conductivity of air ($k_a = 0.026\ W\ m^{-1}\ K^{-1}$) [3], k_m is the thermal conductivity of the membrane, equal to 0.44 $W\ m^{-1}\ K^{-1}$ in the case of PE without submicron particles [4] and calculated by the Bruggeman model in the case of PE with SiO_2-submicron particles [5,6], ε_s is the emissivity of human body ($\varepsilon_s = 1$), ε_a is the emissivity of the environment ($\varepsilon_a = 1$), h_c is the convective heat transfer coefficient ($h_c = 3\ W\ m^{-2}\ K^{-1}$), σ is the Stefan-Boltzmann constant ($\sigma = 5.67 \times 10^{-8} W\ m^{-2}\ K^{-4}$), h_{mc} is the thickness of the microclimate ($h_{mc} = 2\ mm$) and h_m is the thickness of the membrane.

The membrane's reflectance (ρ), transmittance (τ), and absorbance (α) are calculated by the formula:

$$\eta = \frac{\int_{\lambda_{min}}^{\lambda_{max}} E_\lambda . \chi_\lambda . d\lambda}{\int_{\lambda_{min}}^{\lambda_{max}} E_\lambda d\lambda} \tag{10}$$

where E_λ is the human body radiation and χ_λ is one of the R, T or A coefficients at the wavelength λ. The integration is done over the wavelength range [λ_{min} = 5 μm – λ_{max} = 15 μm].

According to the Kirchhoff law, the reflectance, transmittance and emittance of the membrane are connected by the formula

$$\varepsilon_m = \alpha = 1 - \rho - \tau \tag{11}$$

References

1. J. K. Tong, X. Huang, S. V. Boriskina, J. Loomis, Y. Xu, and G. Chen, ACS Photonics 2, 769 (2015).
2. American Society of Heating, *2009 ASHRAE Handbook -- Fundamentals (SI Version): (Includes CD in Dual Units.* (2009).
3. Y. Yamashita, H. Yamada, and H. Miyake, J. Text. Eng. 54, 111 (2008).
4. I.-L. Ngo, S. Jeon, and C. Byon, International Journal of Heat and Mass Transfer 98, 219 (2016).
5. J. K. Carson, S. J. Lovatt, D. J. Tanner, and A. C. Cleland, International Journal of Heat and Mass Transfer 48, 2150 (2005).
6. Y. Hwang, J. K. Lee, C. H. Lee, Y. M. Jung, S. I. Cheong, C. G. Lee, B. C. Ku, and S. P. Jang, Thermochimica Acta 455, 70 (2007).
